# Clinical Source Data Production and Quality Control in Real-world Studies: Proposal for Development of the eSource Record System

**DOI:** 10.2196/42754

**Published:** 2022-12-23

**Authors:** Bin Wang, Junkai Lai, Feifei Jin, Xiwen Liao, Huan Zhu, Chen Yao

**Affiliations:** 1 Peking University Clinical Research Institute Peking University First Hospital Beijing China; 2 Institute of Automation Chinese Academy of Sciences Beijing China; 3 Trauma Medicine Center Peking University People's Hospital Beijing China; 4 Key Laboratory of Trauma treatment and Neural Regeneration Peking University Ministry of Education Beijing China; 5 National Center for Trauma Medicine of China Beijing China; 6 Hangzhou LionMed Medical Information Technology Co, Ltd Hangzhou China; 7 Hainan Institute of Real World Data Qionghai China

**Keywords:** electronic medical record, electronic health record, eSource, real-world data, eSource record, clinical research, data collection, data transcription, data quality, interoperability

## Abstract

**Background:**

An eSource generally includes the direct capture, collection, and storage of electronic data to simplify clinical research. It can improve data quality and patient safety and reduce clinical trial costs. There has been some eSource-related research progress in relatively large projects. However, most of these studies focused on technical explorations to improve interoperability among systems to reuse retrospective data for research. Few studies have explored source data collection and quality control during prospective data collection from a methodological perspective.

**Objective:**

This study aimed to design a clinical source data collection method that is suitable for real-world studies and meets the data quality standards for clinical research and to improve efficiency when writing electronic medical records (EMRs).

**Methods:**

On the basis of our group’s previous research experience, TransCelerate BioPharm Inc eSource logical architecture, and relevant regulations and guidelines, we designed a source data collection method and invited relevant stakeholders to optimize it. On the basis of this method, we proposed the eSource record (ESR) system as a solution and invited experts with different roles in the contract research organization company to discuss and design a flowchart for data connection between the ESR and electronic data capture (EDC).

**Results:**

The ESR method included 5 steps: research project preparation, initial survey collection, in-hospital medical record writing, out-of-hospital follow-up, and electronic case report form (eCRF) traceability. The data connection between the ESR and EDC covered the clinical research process from creating the eCRF to collecting data for the analysis. The intelligent data acquisition function of the ESR will automatically complete the empty eCRF to create an eCRF with values. When the clinical research associate and data manager conduct data verification, they can query the certified copy database through interface traceability and send data queries. The data queries are transmitted to the ESR through the EDC interface. The EDC and EMR systems interoperate through the ESR. The EMR and EDC systems transmit data to the ESR system through the data standards of the Health Level Seven Clinical Document Architecture and the Clinical Data Interchange Standards Consortium operational data model, respectively. When the implemented data standards for a given system are not consistent, the ESR will approach the problem by first automating mappings between standards and then handling extensions or corrections to a given data format through human evaluation.

**Conclusions:**

The source data collection method proposed in this study will help to realize eSource’s new strategy. The ESR solution is standardized and sustainable. It aims to ensure that research data meet the attributable, legible, contemporaneous, original, accurate, complete, consistent, enduring, and available standards for clinical research data quality and to provide a new model for prospective data collection in real-world studies.

## Introduction

### Background

Real-world data (RWD) are the data relating to patient health status and delivery of health care routinely collected from a variety of sources [1]. Real-world evidence (RWE) is clinical evidence regarding the use and potential benefits or risks of a medical product derived from analysis of RWD [1]. A real-world study (RWS) collects RWD in a real-world environment and obtains RWE of the use value and potential benefits or risks of medical products through analysis. There is considerable interest in the use of RWD to generate RWE to support regulatory decisions regarding the effectiveness of medicines. However, large data sets of uncertain quality and origin, lack of readily available analytical tools, and lack of sufficiently methodologically proficient researchers can lead to flawed study designs and analyses that yield incorrect or unreliable conclusions [1]. Although important advances are being made in the field of methodologies to access RWD, these factors are not sufficient to fully overcome the fundamental issues of confounding, data quality, and bias [1]. The US Food and Drug Administration (FDA) states that gaps in RWD sources need to be addressed first, as electronic health record (EHR) and medical claims data may not capture all the data elements needed to answer questions of interest [2]. Another important challenge is the difficulty in connecting or integrating the various data sources that provide information about individual patients [3]. The review by Grimberg et al [4] outlines the RWD challenge radar and summarizes the challenges and risks of using RWD from 3 perspectives (organizational, technological, and people-based), for example, inefficient data collection, lack of data quality control, diversification of data standards, and facing data compliance issues [4].

In clinical studies, source data refer to all the information in the original records or their certified copies, including clinical findings, observation results, and records of other relevant activity that are necessary for the reconstruction and evaluation of the trial [5]. eSources are data that are originally recorded in an electronic format. An eSource generally includes the direct capture, collection, and storage of electronic data (eg, electronic medical records [EMRs], EHRs, or wearable devices) to simplify clinical research [6]. It can improve data quality and patient safety and reduce clinical trial costs. However, owing to many challenges [7], such as limited interoperability of EMRs and electronic data capture (EDC) systems, unstructured data (eg, researcher notes or comments), and the need for some data (eg, research-specific data that are not included in the EMR) to be manually transcribed and treated, accessing and correcting the source data in real time during data collection can be slow. Despite the existence of several FDA guidelines [6,8] and European Medicines Agency guidelines [9], the development, implementation, and evaluation of EMR-specific electronic resource solutions are limited. The ideal eSource technology will be able to completely bypass EDC data input, capture the source data directly from EMR, and transmit it to an electronic case report form (eCRF). In the past 10 years, a variety of eSource solutions have been developed, evaluated, and improved [10-12]. There has been some eSource-related research progress in relatively large projects, such as the OneSource project, Electronic Health Records for Clinical Research project, and Seventh Framework Program–Translational Research and Patient Safety in Europe project [13-15]. However, most of these studies focused on technical explorations to improve interoperability among systems to reuse retrospective data for research. Few studies have explored source data collection and quality control during prospective data collection from a methodological perspective.

The attributable, legible, contemporaneous, original, accurate, complete, consistent, enduring, and available (ALCOA+) standard has been adopted in the guidelines and industry norms of many regulatory agencies and has become a recognized quality standard for clinical research data [16]. The FDA and European Medicines Agency use ALCOA+ as a guide for protecting data integrity. The World Health Organization has also issued *Guidance on Good Data and Record Management Practices* based on this principle [17]. Good documentation practices and data integrity are integral elements of data management and the foundation of any quality system. The ALCOA+ principles are the cornerstone of good documentation practices and apply to both electronic and paper data. At the good clinical practice (GCP) seminar held in 2020 [18], the FDA and the UK Medicines and Healthcare Products Regulatory Agency proposed new global challenges to data integrity, such as the use of eSource, EHR, and other patient data repositories as RWD sources. Although regulators in different countries have recently issued guidance and strategies to enhance data integrity [17,19-22], challenges remain in how to apply this principle in practice to safeguard data integrity in RWD.

Source data verification (SDV) means to check the consistency of data recorded in the database with the source data, and it is a key link in maintaining data accuracy in quality control and evaluating data integrity in on-site verification by regulatory authorities. In China, external access and data sharing are not possible owing to the sensitivity of medical data. Therefore, SDV is usually performed using a printed and signed copy of EMRs. Owing to the inability to reconcile hospitals’ concerns about the privacy of patient medical data and researchers’ needs for data transparency, the transformation and upgrade of EMR systems by existing medical system providers still cannot meet the requirements of clinical research [23].

Data integrity in clinical research is a critical issue for both the health care system and research community, and the consequences of not maintaining data integrity can be severe, including regulatory violations, need for additional research, reputational damage, and paper retraction. A retraction analysis of clinical studies has shown that it is important to develop processes that enhance the detection of defective products in their respective likely environments [24]. After the China National Medical Products Administration issued the most stringent data verification requirements in 2015, a total of 80% of studies on new drug applications were withdrawn [25]. In 2016, a foreign researcher published an article in the *British Medical Journal* claiming that 80% of China’s clinical trial data were fraudulent, which brought great reputational damage to China’s clinical research field [26]. In 2018, our team presented an opinion in the *British Medical Journal’s* international community on how to protect the accuracy of clinical trials in China [23]. We propose a solution to improve the integrity of clinical research data in China by using the hospital clinical research source data management platform and source data management process architecture. A clinical source data management platform for electronically synchronizing and storing all study-related source data not only protects the integrity and accuracy of study data but also facilitates SDV by internal or external supervisors, auditors, and researchers themselves.

Currently, there are many medical standard–setting organizations and institutions dedicated to the interoperability of EMRs to support RWD collection and analysis [27]: (1) diverse common data models, such as Clinical Data Interchange Standards Consortium (CDISC) Study Data Tabulation Model, Observational Medical Outcomes Partnership [28], FDA Sentinel [29], and National Patient-Centered Clinical Research Network [30], and (2) data exchange standards, such as the Health Level Seven Fast Health Interoperability Resources [31], CDISC operational data model (ODM) [32], and openEHR. However, these standards are not yet able to address all of China’s needs, and much work is still needed before they can be implemented. Improving medical data interoperability cannot fundamentally solve the problem of data integrity. In addition, except for some large state-funded projects, most of the research is limited to case studies, thus failing to propose a general theoretical method, and very few studies can achieve the transformation from theory to results, real implementation, and promotion.

In the previous study by our research group, a hospital clinical research source data management platform and source data management process architecture were proposed [33]. The core factor for improving the quality of research data is the promotion of the electronification of clinical research source data; in particular, there is a need to break through the barriers between the clinical diagnosis and treatment data and the clinical research system. Subsequently, the research group explored an RWD collection mode based on hospital informatization and verified it using an RWS of medical devices [34]. The study found that when natural language processing (NLP) was used, the completion time was reduced by 90% compared with methods that relied on manual input [34].

### This Study

This study is an in-depth exploration based on previous results. Using the eSource concept, we designed a source data collection method for clinical medicine that is suitable for RWSs, meets the data integrity standards for clinical research, and realizes electronic transmission from source data to clinical research data. We developed a piece of software using the proposed method and applied it to an RWS to verify its feasibility [35].

## Methods

### Design and Optimization of the Source Data Collection Method

On the basis of the task decomposition steps proposed by the ALCOA+ principles, we designed the method by referring to the eSource logical architecture diagram proposed by TransCelerate BioPharm Inc [7], RWD, eSource-related regulatory guidelines [2,3,6,8,9,36-38], and the research group’s previous experience. In the process of designing and optimizing the method, the members of the research team and experts in related fields extensively solicited, communicated, and discussed suggestions through focus groups and expert consultations. Experts in related fields included the big data company’s technical staff (product managers, front-end and back-end developers, etc), clinical trial personnel in different roles (principal investigators, clinicians, project managers, clinical research associates [CRAs], clinical research coordinators [CRCs], data managers [DMs], etc), hospital information personnel, experts from the drug regulation department, and so on. Using this method, we cooperated with a big data company to develop the eSource record (ESR) system. The ESR system is a piece of software that is implemented in a hospital in addition to an EMR system and a trial management system (such as an EDC system). It can be considered as a connecting bridge between an EMR system and EDC system. To create a complete set of clinical research source data solutions, we invited experienced experts from EDC companies in different clinical trial roles to provide their input. Guided by GCP principles, we addressed the issue of data connection between an ESR and EDC system.

### Ethics Approval

This study was conducted in accordance with the Declaration of Helsinki. Ethics approval was obtained from the Peking University’s institutional review board (IRB00001052–21081).

### Task Decomposition of ALCOA+ Principles in Source Data Collection Methods

#### A—Attributable

It can be very simply summarized as that the person who performs the data-related task must be the person who performs the task. For any operation, an ESR system should reliably track only the user who created, modified, or deleted the data. The entire process from the source data to the final analysis data set should be clearly recorded. The producer of each source datum, date and time it was produced, relationship between the source datum and its attributor (such as the patient), reason for the modification of the source data and related evidence, and so on should be clearly reflected in the quality of the source data in the chain of custody.

#### L—Legible

The data should be readable and understandable and clearly show the sequence of steps or events the data have gone through. It should cover the terminology mapping function and use CDISC standard terminology as much as possible. The ESR solution has designed but has not yet implemented a standard terminology input mode that can correct mistakes in terminology use for documentation.

#### C—Contemporaneous

Data activities should be time-stamped, and the time of occurrence should be recorded. ESR can use recording and other functions to retain the voice recording of the physician during the consultation of the patient and to realize the real-time collection of source data.

#### O—Original

All the initially captured data must be retained; they should not be replaced or deleted. ESR should preserve the source data to ensure the originality of the original record. It should only back up the data in the hospital and the data outside the hospital, without any data cleaning operations, to ensure the originality of the certified copy. The certified copy of the original record shall be verified as having all the same attributes and information as the original record and shall be certified according to the dated signature. All recording files and various source files, such as pictures uploaded during optical character recognition (OCR), will be retained.

#### A—Accurate

Data input, storage, and maintenance should be accurate and effective. ESR conducts quality control on data through multiple links, such as electronic system verification; clinician medical record writing; data encryption; transmission; management process; and CRC, CRA, and DM verification, to ensure the accuracy of data.

#### C—Complete

The data should have a traceable audit trail to prove that nothing has been deleted or lost. ESR can highlight the uncollected indicators in the medical record writing promptly to remind the clinician to record the research indicators completely. It can also check the integrity of the data through the data quality control link and return it to the clinician. The CDISC ODM data standard format for eCRF is not widely or professionally implemented in China because it is not a requirement for drug submissions. However, EDC companies have started to implement CDISC ODM as a method of data exchange. Currently, the ESR solution uses the CDISC ODM as a method of data exchange with EDC companies. However, certain features, such as the audit trail feature, are not implemented consistently by different EDC companies; therefore, the CDISC ODM format used by the ESR will vary based on the partnered company.

#### C—Consistent

Regardless of where the data are accessed from, they should be displayed consistently. The ESR can verify the consistency of source data and research data through CRC verification, and the CRA and DM can perform traceability verification, raise data questions, and further check consistency.

#### E—Enduring

Records and information should be accessible and readable for the entire period that they may be needed, possibly decades after they are recorded. The ESR can prevent data loss in the event of interruption through system backup. Verified electronic record backup should be provided to ensure disaster recovery.

#### A—Availability (Available)

All applicable personnel responsible for reviewing or operating procedures should access files and records in a readable format. The ESR can output source data in an appropriate format for reference through processes such as data processing, data structuring, and data standardization.

## Results

### Description of the Source Data Collection Method

#### Overview

**Figure 1:**
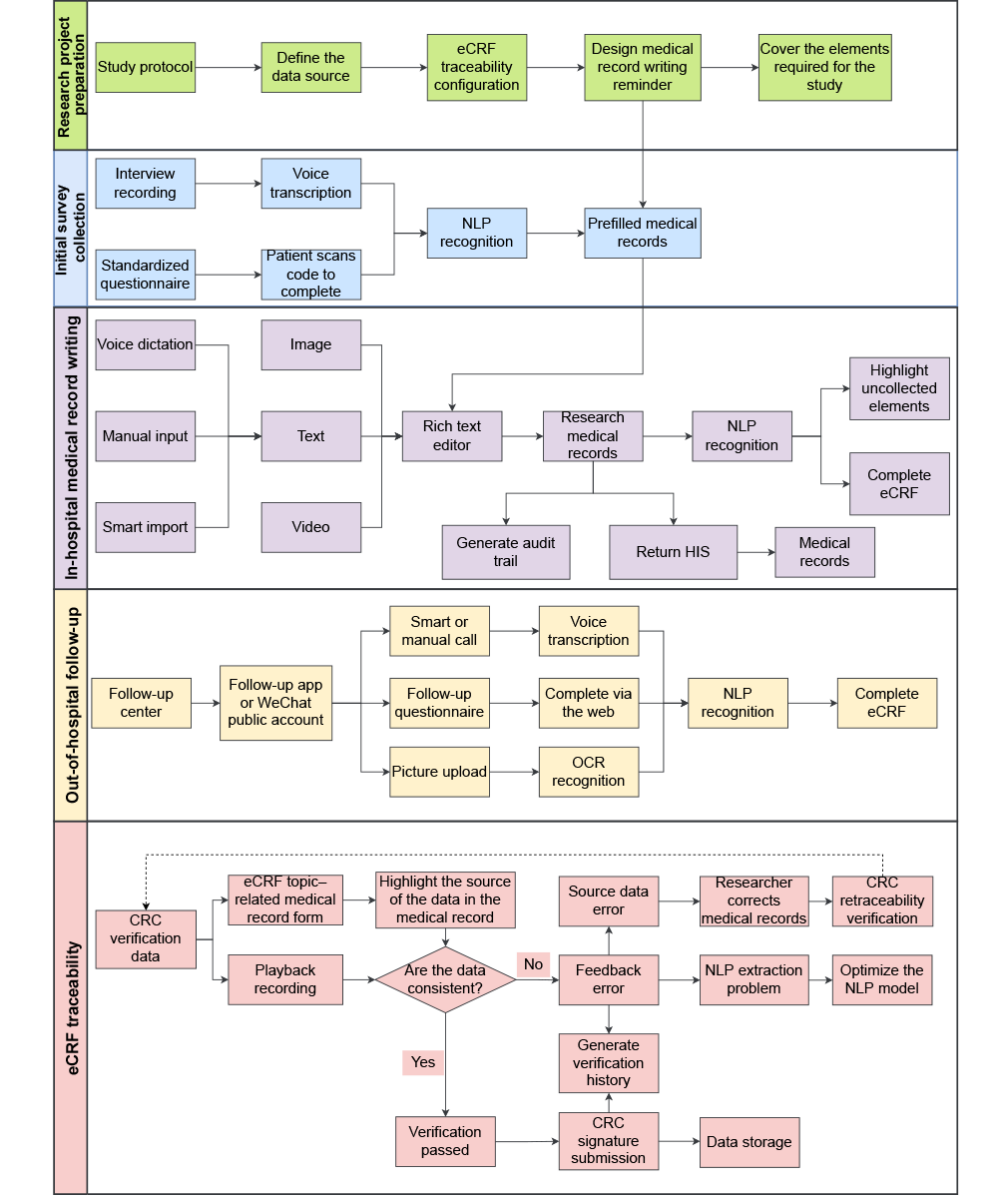
Flowchart of the source data collection method. CRC: clinical research coordinator; eCRF: electronic case report form; HIS: hospital information system; NLP: natural language processing; OCR: optical character recognition.

The method includes 5 steps: research project preparation, initial survey collection, in-hospital medical record writing, out-of-hospital follow-up, and eCRF traceability. A flowchart of this method is shown in Figure 1.

#### Research Project Preparation

In the preparation stage of a research project, such as a randomized controlled trial, the researcher needs to determine the research plan. The plan should clearly define the data elements that need to be collected; determine the data source and data type; and define the source data collection method, time of data collection, and personnel who will collect the data. According to the data sources, research data can be divided into data collected by the hospital electronic system, additional data collected during research, and data collected outside the hospital. The data collected by the hospital EMR system are medical data generated by the patient during the hospital visit or hospitalization; these may include EMR data, medication data, and medical insurance data. Research-specific data are additional data collected in the hospital according to the needs of the research project; these may include the recording of certain additional index data during surgical operations. Data collected outside the hospital are research-related data that are generated after the patient is discharged and may include follow-up data such as adverse events. Research data are divided into 2 data types: structured data and unstructured data. An eCRF can be designed based on the research plan. eCRF topics can be associated with the EMR form to configure the traceability path of different eCRF topics. For example, demographic data in eCRF can be traced back to the admission record form in the EMR. However, routine medical records do not contain certain necessary research-specific data, such as scale scores. Therefore, after completing the eCRF traceability configuration, clinicians can design medical record writing prompts and rules for the eCRF that conform to clinical habits and meet their data collection requirements, to cover the elements required for research and standardize the EMR recording process among different clinicians.

#### Initial Survey Collection

The collection of research data can be divided into initial survey collection and in-hospital medical record writing. During the collection of initial survey data, the clinicians’ workload when writing EMRs can be reduced with the use of voice transcription and NLP technology and by allowing patients to fill in some of the information. For example, as the hospital admission medical record (basic information and past history) involves few complicated medical terms, a standardized questionnaire can be created, and the patient can scan a QR code to access it and complete it. The data for the main complaint and current medical history sections can be collected by clinicians through a traditional medical history interview; then, the dialogue between the clinician and patient is transcribed into text in real time using voice transcription technology, and information such as symptoms, medicines, time, disease diagnosis, and so on are analyzed and extracted using NLP technology. Finally, this information is prefilled into the medical records.

#### In-Hospital Medical Record Writing

In-hospital medical record writing is the process by which clinicians write medical records according to the research medical record template. The rich text editor allows clinicians to record information such as text, pictures, and videos. Data input methods for text-type information are divided into voice dictation, manual input, and intelligent import. Intelligent import technology refers to the automatic or semiautomatic transmission of clinical data from one data field or system to another data field or system (eg, via copy and paste, autofill, barcode scanning, or image OCR). Clinicians further process and sort machine-prefilled medical records and record the source data from the medical encounter in a timely and complete manner to create research medical records. NLP technology can highlight elements that are not collected by clinicians in real time as they write medical records. For medical records that have been completed and submitted with signatures, NLP can extract research data from the background and automatically complete the eCRF. The system can track all the revisions that clinicians have made in the research medical records for verification. As the research medical records were recorded by the software we designed, to connect them to the hospital’s EMR system, we transferred all the research medical records back to the EMR in the form of documents to create a medical record. This avoids the need for clinicians to complete the medical records twice in the 2 systems, because records completed in accordance with research requirements include more information and can meet the requirements for medical records.

#### Out-of-Hospital Follow-up

Through the follow-up center, clinicians can use follow-up apps or official WeChat accounts to collect out-of-hospital data needed for research. After setting the trigger conditions for the follow-up start time, frequency, and format, the system can automatically send the follow-up questionnaire to the patient, who can complete the questionnaire via the web. It is also possible to use smart or manual calls for follow-up questions; this allows patients to participate in question-and-answer dialogues, which the system can then transcribe into text. For laboratory examinations and imaging performed outside the hospital, the system offers file upload functions and OCR of pictures and text. After summarizing these different forms of follow-up data, NLP extracts these contents and enters them into the eCRF.

#### eCRF Traceability

In the steps mentioned previously, NLP automatically extracts research data from EMRs and out-of-hospital follow-up records and uses them to complete the eCRF. This can greatly reduce the workload of the CRC, who will no longer need to manually complete the eCRF and can focus on data verification. As the researcher completed the configuration of the eCRF topic and medical record form during the preparation phase of the research project, when the CRC opens the eCRF traceability function, the EMR written by the physician and the eCRF form will be displayed on the screen at the same time. When the CRC clicks on the eCRF topic, the system can automatically scroll to locate and highlight the position of answers in the source file. The CRC can also be led to the sources of data by playing the recordings maintained in the system (eg, recordings of consultations or medical records created by physicians using voice input). Then, the CRC can check whether the data recorded in the source file are consistent with the data extracted by NLP. If all the data pass this check, the CRC will sign and submit the certification to complete the data storage. If the CRC finds inconsistent data, they can provide feedback indicating the presence of an error. For problems related to NLP extraction, the CRC can make manual corrections and provide feedback to the technicians to optimize the NLP model. If there is an error in the source data, such as an error introduced by the physician during the writing of the medical record, feedback is provided to the clinician, who will correct the content of the EMR and resubmit it. The NLP will extract it again, and the CRC will recheck the issue. The system will record the history of all the CRC verifications.

### Data Docking Between the ESR and EDC

**Figure 2:**
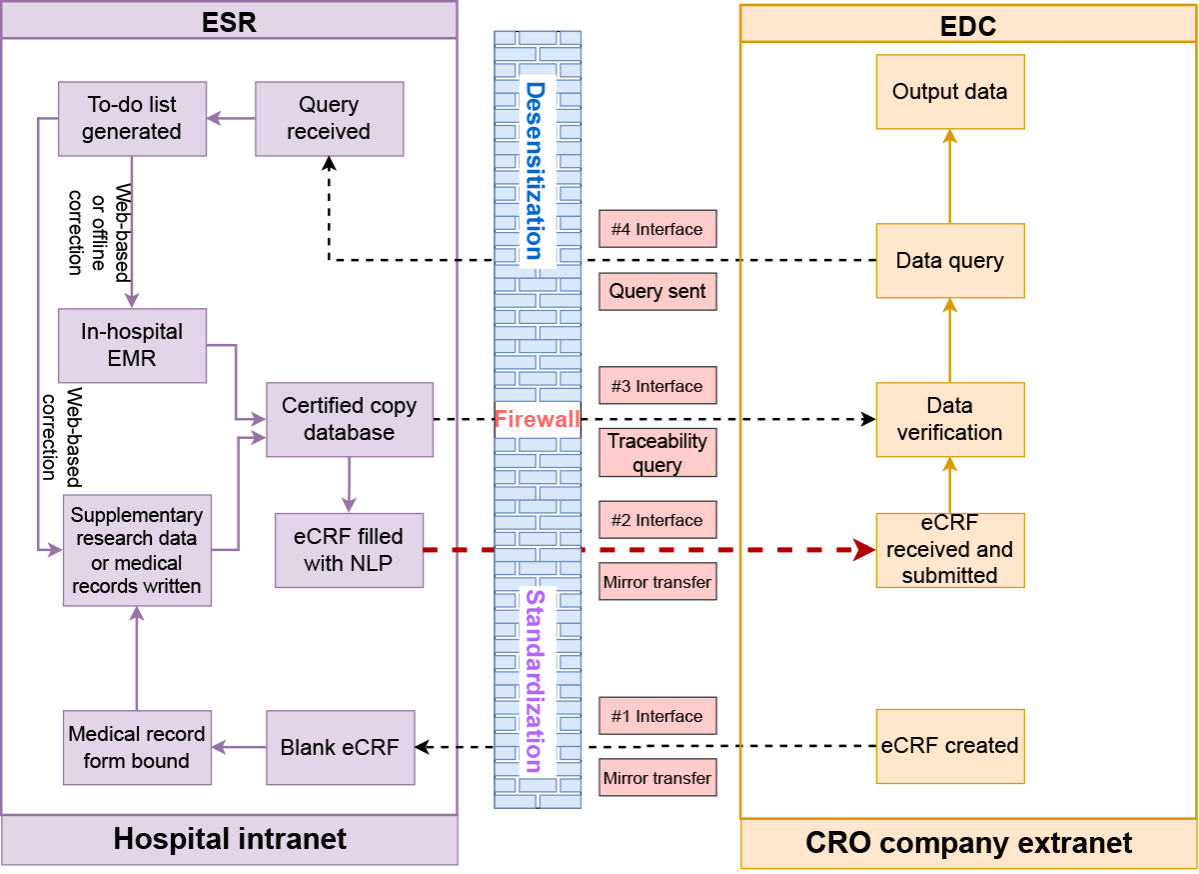
Data docking between the eSource record (ESR) and electronic data capture (EDC) systems. CRO: contract research organization; eCRF: electronic case report form; EMR: electronic medical record; NLP: natural language processing.

Although the ESR that we designed can theoretically integrate EDC functions, as the implementation of this method requires a transition phase and integration of the CDISC Clinical Data Acquisition Standards Harmonization data standard used by EDC, we will discuss the process of creating a data connection between the ESR and EDC (Figure 2). First, clinicians and data administrators enter the EDC to create an empty mirror eCRF based on the CDISC data standard and pass it to the ESR system. The eCRF is decomposed using the abovementioned source data collection method, and the research medical record writing requirements and configuration traceability paths corresponding to different data collection points are designed. The ESR integrates out-of-hospital data and process supervision source data. Clinicians need to write their medical records only on the ESR, and the ESR can automatically synchronize data with the in-hospital EMR system. The intelligent data acquisition function of the ESR will automatically complete the empty eCRF to create an eCRF with value. Then, the in-hospital, out-of-hospital, and process supervision data can be backed up to form a certified copy database. The certified copy database needs to undergo data management to create a clinical research database. Then, the CRC can use ESR functions such as audio replay, picture review, and highlight traceability desensitization data to track the clinical research database in real time to verify the valued eCRF data and submit it to create a confirmed eCRF. By transmitting data that mirror the eCRF values, the EDC receives and submits the eCRF. When the CRA and DM conduct data verification work, they can query the certified copy database through interface traceability and send data queries. The data queries are transmitted to the ESR through the EDC interface. If the source data are incorrect, they will be generated in the ESR system, and the corresponding researchers will be notified to correct the medical records. For the ESR system to send data queries to the EMR system, the EMR company adds a module to visualize the ESR interface; therefore, all notifications or queries can be directly handled within the EMR. Researchers can choose to correct the source data via the web or offline in the EMR system. If some supplementary research data are recorded through the ESR system, the correction of source data can be completed via the web in the ESR system. After completing the source data correction, ESR automatically synchronizes the source data to the certified copy database again, re-extracts the research data, completes the eCRF, and finally passes the eCRF values to the EDC system through the interface. The data verification process described previously is performed until the verification is completed. When all data verification is completed, the principal investigator can sign and lock the database to secure the research and analysis data that can be used by statisticians.

### Data Standard Transformation From RWD to Research Data

Research data need to communicate with source data systems if data integrity is to be met. There are 2 approaches mentioned in the relevant FDA report [8], including interoperable systems and fully integrated systems. Data integrity can be fully guaranteed only if clinical researchers are allowed to enter study data directly into the EHR (fully integrated system). In contrast, interoperable systems usually only pass a portion of the data that are mature and standardized. EDC and EMR systems will interoperate through the ESR. The EMR and EDC systems transmit data to the ESR system through the data standards of Health Level Seven Clinical Document Architecture and CDISC ODM, respectively. When the implemented data standards for a given system are not consistent, ESR will approach the problem by first automating mappings between standards and then handling extensions or corrections to a given data format through human evaluation. The ESR can receive the familiar document format in the EMR and eCRF fields through EDC, provide writing suggestions in the EMR document, and send the suggestions back to the EMR system. The ESR process includes 5 steps, as shown in Textbox 1 [39,40].

**Steps in the eSource record (ESR) method.**

**Step 1**

- Electronic data capture sends the eCRF and electronic medical record sends the patient clinical form to the ESR system. The source data collection module of the ESR system will be responsible for the annotation of electronic medical records, whereas the data transcription module of the ESR system will be responsible for locking the electronic case report form (eCRF) field to capture text segments of source data, complete the eCRF, and generate a traceability interface for clinical research coordinator review.

**Step 2**

- The second step involves modeling the research data set and generating labels. Structured data are directly mapped to the Clinical Data Interchange Standards Consortium (CDISC) model. Unstructured data do not have a widely used intermediate layer and do not consider the Observational Medical Outcomes Partnership model but directly converts to the CDISC model. The process of converting unstructured data to research data requires annotating the text and extracting the relevant content using natural language processing models.

**Step 3**

- The third step involves model training and extraction of entities and relationships between entities. Regarding entity extraction, the Chinese-named entity recognition model of bidirectional encoder representation from transformers, bidirectional long short-term memory neural networks, and conditional random fields are used.

**Step 4**

- The fourth step involves the generation of research-specific term database. The research-specific term database refers to the mapping library between the actually extracted terms in the tags and the standard terms. The establishment of a research-specific term database requires the extracted tags, CDISC operational data model code lists, and international standard terms (such as International Classification of Diseases 10th Revision).

**Step 5**

- The final step is related to normalization rules after entity extraction and before completing the eCRF. The output of the natural language processing model mainly has 2 tables, including the list of all the extracted label values (entity table) and the list of relationships between entities (entity relationship table). The first task was to assign each entity label with a standard value and standard label type using a research-specific term database. The second task was to convert the entity relationship table to a single record based on the domain.

### Case Verification

**Figure 3:**
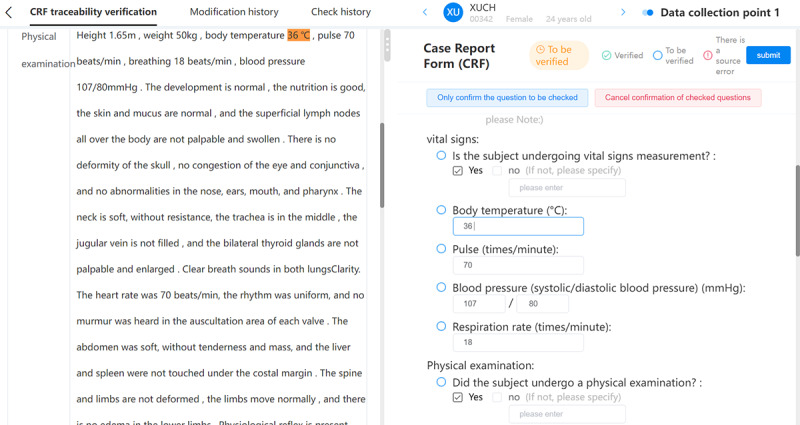
The clinical research coordinator interface for data traceability verification in the eSource record.

**Figure 4:**
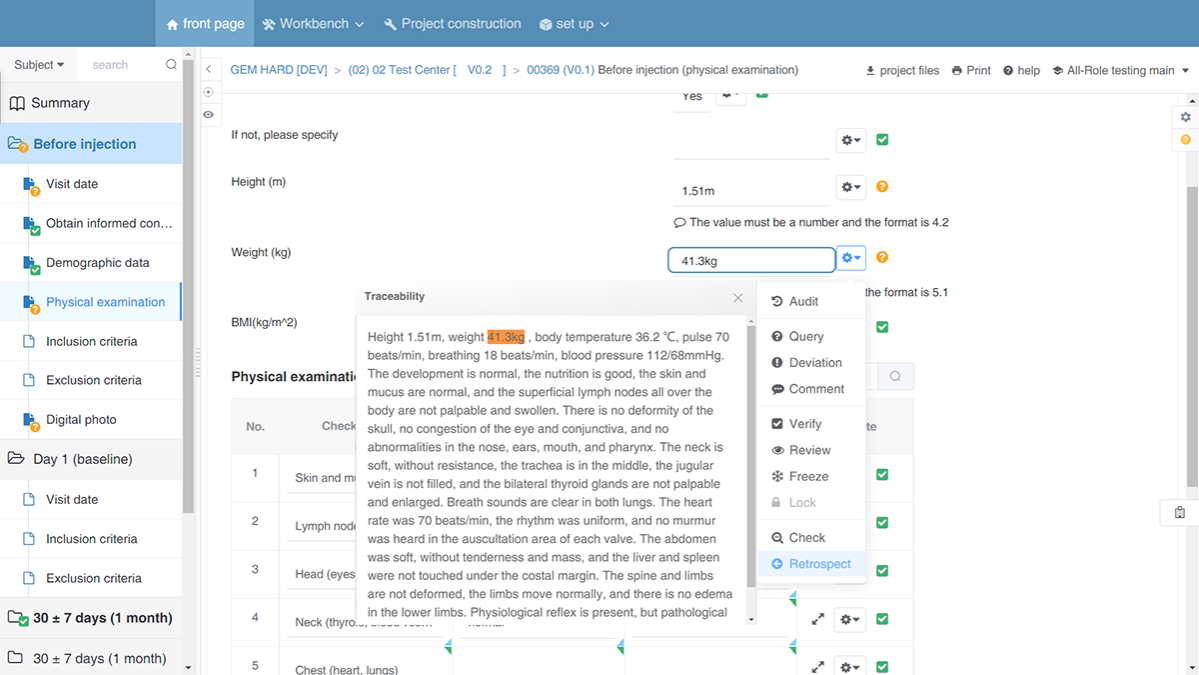
Interface for traceability operation in electronic data capture.

In 2021, we selected an RWS to evaluate the effectiveness and safety of cosmetic medical equipment (cross-linked glucan) for chin augmentation in the Boao Lecheng pilot zone. The interface that allows the CRC to use the ESR for data traceability verification is shown in Figure 3. This figure shows the interface under the eCRF traceability verification label. The contents of the outpatient medical records are shown on the left. The eCRF topic is shown on the right. When the mouse stays in the answer box for “body temperature” on the right, the answer “36 ℃” is retrieved from the text related to the physical examination and is highlighted on the left. The operation interface for CRC or DM traceability in EDC is shown in Figure 4. This figure shows the interface under the eCRF traceability verification label. The contents of the outpatient medical records are shown on the right. The eCRF topic is shown on the left. When the mouse stays in the answer box for “body temperature” on the right, the answer “36 ℃” is retrieved from the text related to physical examination and highlighted on the left. When you click the *retrospect* function in the drop-down menu, the original record will pop up and the text will be highlighted. This figure shows the body weight from the physical examination part of the outpatient medical record. Details about case verification are available in our previously published study [41]. The preliminary evaluation shows that in the clinical medical environment, the ESR-based eSource method can improve the efficiency of source data collection and reduce the workload required to complete data transcription [41]. Since the initial verification in this RWS, we have collaborated with many other projects for more extensive verification. These pilot projects have begun the process of deploying the tool in hospitals and will start soon. A project currently using the ESR is the RWS on the safety and efficacy of injectable cartilage-regenerating collagen fillers for the treatment of cartilage damage. At the same time, we have initiated collaboration with medical system providers to develop a way to integrate this tool with EMRs. These rich cases will provide a large amount of data for evaluating the value of the tool and promoting the development of clinical research in China.

## Discussion

### Principal Findings

Although previous studies have applied Fast Health Interoperability Resources or openEHR standards to interoperability cases to serve as experiential references [42,43], clinical research data include research-specific data that are not routinely recorded in the EMR. In addition, the free-text data recorded by the physician in medical records are not adequate to meet these data standards, and additional data must be extracted using NLP technology. Wehrle et al [44] created a data control framework to support high-quality RWSs using the NeuroTransData system to collect data from registry databases in multiple disease areas. Although this study covers the data cycle from input to analysis, the limitation is that SDV cannot be performed on all data. The method randomly sampled data from only 10 patients per year and investigated the consistency of source data documentation in EHRs, practice management software systems, and NeuroTransData registry. The study by Chatzidimitriou et al [45] illustrated the challenges and solutions for collecting and analyzing RWD using the chronic lymphocytic leukemia database as an example. The researchers proposed a unified data management framework to allow the collection of homogeneous high-quality data sets and the connection of multiple forms of biological and medical information. The main limitation of this framework is that it does not include quality control measures for SDV. Abdolkhani et al [46] discussed wearable health data solutions for RWD quality control in a workshop format. However, this study only proposed 5 general solutions for the attributes of health data and has not yet formed a complete theoretical framework.

Our study explores ways to implement eSources when conducting clinical research in the current medical environment. Our ESR solution provides novel options for addressing these challenges. It is simple and can be easily implemented, without requiring changes in the medical system. By managing data from different sources, the ESR can meet the requirements of data standards and provide traceability for verification. It can address the scientific research pain points of clinicians in the following ways: (1) clinicians can formulate medical record writing rules consistent with their clinical habits that comply with the research plan; (2) NLP tools can be integrated into web-based operations, allowing clinicians to extract text information without any experience in programming; (3) on the basis of the initial model and the corpus marked by clinicians, the model can undergo dynamic learning and optimization; (4) after the model meets the expected requirements, it can automatically label and extract information, which solves the problems related to traditional manual data collection; and (5) a feedback loop is established for clinicians’ case writing to improve subsequent medical record writing specifications and ultimately ensure high quality of research data.

### Limitations

Just as the data standards and use communities of different data models are different, the ESR will inevitably face some challenges in its follow-up, such as how to integrate with the EMR as a lightweight plug-in to improve clinicians’ acceptance when connecting to EDC and health information systems produced by different manufacturers. The biggest hurdle is that China’s hospital medical record system vendors built their systems long before industry standards were implemented, resulting in lack of standards that could be used for data exchange. Finally, all the challenges of implementing ESR presented by different stakeholders are not fully addressed in this study.

### Conclusions

The main contribution of this study is the creation of a source data collection method that realizes a new eSource strategy. The ESR solution aims to meet the ALCOA+ standards for clinical research data integrity and provide a new model for prospective data collection in RWSs. Unlike other attempts to solve data interoperability, which are not always applicable, the ESR that we proposed was designed in accordance with the GCP principle, which is standardized and sustainable. The integration of NLP technology into the ESR improves its flexibility, thus increasing the ease with which clinicians can extract research data.

## Abbreviations

ALCOA+: attributable, legible, contemporaneous, original, accurate, complete, consistent, enduring, and available
CDISC: Clinical Data Interchange Standards Consortium
CRA: clinical research associate
CRC: clinical research coordinator
DM: data manager
eCRF: electronic case report form
EDC: electronic data capture
EHR: electronic health record
EMR: electronic medical record
ESR: eSource record
FDA: Food and Drug Administration
GCP: good clinical practice
NLP: natural language processing
OCR: optical character recognition
ODM: operational data model
RWD: real-world data
RWE: real-world evidence
RWS: real-world study
SDV: source data verification

## Data Availability

Data sharing is not applicable to this paper, as no data sets were generated or analyzed during this study.
